# Mel – Personal Reminiscence^†^

**DOI:** 10.3389/fped.2014.00049

**Published:** 2014-07-03

**Authors:** H. William Taeusch

**Affiliations:** ^1^Department of Pediatrics, San Francisco General Hospital, University of California San Francisco, San Francisco, CA, USA

**Keywords:** Avery, lung, pulmonary, surfactants, neonatology, medical research, lungs

On July 1, 1970, I started my fellowship in neonatology under the tutelage of Mary Ellen Avery. I entered her lab in the MacIntyre Building at McGill University in Montreal. There were no other fellows or techs working in her lab at the time. The lab was spacious but sparsely furnished. In one corner was a pneumatic surface balance, hand-built, from the design of John Clements. A lab book lay on the bench with entries dated June, 1970, by Bob Kotas, my predecessor, who had neatly recorded data on lungs from fetal rabbits. I was alone and knew no one in the building. Mel’s office was several miles away at Montreal Children’s Hospital where she was, surprisingly, the new Chief of Pediatrics (An American! A Woman!).

She was readily accessible in her hospital office but came to the lab only on Wednesday afternoons. There we sat nose to nose for 3 h while she reviewed my week’s work. Not yet trained in the academic art of self-promotion, I once told her in three brief sentences of my past week’s efforts. She waited for more and then looked startled when I remained quiet. With her usual candor she said that after she returned to her office at 5 p.m., by the end of her workday, she would have completed more, much more, than I had managed to do in a week. In heated response I blatted out all of my week’s successes and failures in extenso. She smiled and said, “Well that’s better. You not only have to do well, you have to show that you are doing well.” (Figure 1).

**Figure 1:**
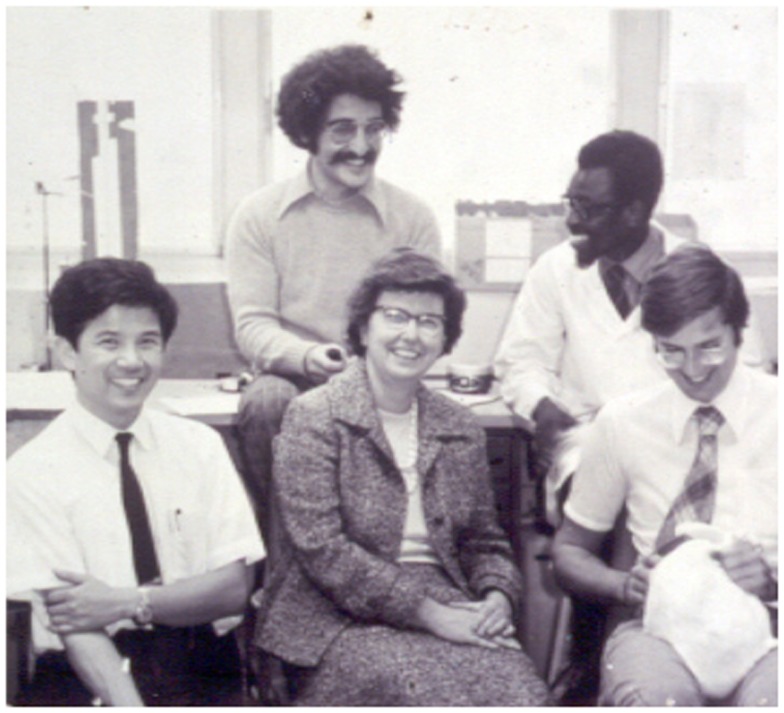
**Montreal lab, about 1970**. Arrayed around Mel from left to right are Nai San Wang, Izzy Wyszogrodski, Kwabena Kyei-Aboagye, and the author (with the bashful bunny). When the sequence of authorship for a paper arose for discussion, Mel, even though she merited being listed first, said, “Put my name last and I’ll still get all the credit, because nobody can remember the names of you jokers.”

One of my many learned lessons at her knee. Not only has she been my mentor for life, she was generous enough to push me to learn from others with talents in some areas that exceeded her own (Jere Mead, David Bates, Joseph Milic-Emili, Peter Macklem, and John Clements, the first Mel Avery awardee of the Pediatric Academic Societies, Vancouver, 2014). Mel believed that cross-disciplinary collaborative research was key for major developments in the field, long before medical centers dressed up this concept as a novel way to get funding. She was impatient of “me-too” research. She recognized and rewarded those that could produce results fitting her Venn diagram (new, true, and useful). Her curiosity extended from the Eskimos in Baffin Bay to hibernating turtles in Newfoundland to prematurely born sheep in New Zealand. She pored through my novel (about a mother deciding whether to allow surgery on her Down Syndrome newborn with multiple life-threatening anomalies) as if it were an NIH grant application (Even those have more chance of approval these days). She did all this with an irrepressible optimism, a robust sense of humor, and an appreciation of her own foibles. Robert Usher once introduced Mel as unique in her ability to sense which path to choose when she came to a research crossroad.

Her principles colored her feminism. Men and women were unequal – women in medicine had heavier burdens to shoulder – often, kids and husbands. Nonetheless Mel expected the time expended/results achieved ratio to be equal for those she chose to work with her, regardless of gender. Her sole criterion for accepting someone was enthusiasm for the task. When a female trainee was paged to leave a research conference for a sick child, Mel loudly asked, “Where’s her husband?” The under representation of women in medicine, however, affronted Mel’s sense of fairness, and she made sure opportunities were made known to qualified women.

Mel was loyal to a fault, but unaccepting of excuses used to explain an absence of productivity, however measured. My favorite excuse was too much clinical time, until she pointed out that in another division, there were some with more clinical responsibilities, and with a greater research output.

She could blow through thickets of verbiage to find and state the truth. One famous example was a seminar where one of us (a hapless unnamed research trainee with the initials JT) was expounding his research results while Mel held the switch that advanced his slides (remember slides?) on the projector. Impatiently she advanced his slides faster and faster in search of the main point. He stood in front of the rest of us talking faster and faster as his slides flew by on the screen. A larger example was her consultancy to the UN where it was politically correct in some quarters to defend female circumcision on religious principle. Mel called it by its right name – child abuse akin to torture.

Mel’s interests when I first met her were the two subjects I disliked most in medical school: pulmonary physiology and steroid biochemistry. So for a time, alone in her lab I injected rabbit fetuses with glucocorticoids or saline, then recorded pulmonary volume curves on lungs from the prematurely born rabbits. After about 5 months of this, I complained to Mel that I had not yet published a research paper (I was young). Mel hid her amusement, almost completely, and said that she appreciated my enthusiasm. The next week she showed up with a young pathologist, Nai San Wang. She sat him down at a microscope and asked him if he could distinguish fetal lung maturity by looking at the H&E slides from newborn rabbits born by cesarean section at different late days in late gestation. That was easy for any pathologist. But Mel forced him to break down the attributes that determined maturity and we spent the afternoon scoring lung maturity of steroid injected and saline-injected fetal rabbits. The next week we did the same thing for the skin specimens from the same rabbit fetuses. And voila! I helped author a paper that indicated a discordance in maturational rates of fetal skin and lungs (1). Just for good measure she added me as a coauthor on a Scientific American article (2).

Mel’s wisdom included the ability to integrate disparate findings for the benefit of babies. She also had the ability to sweep up skeptics and attract new trainees with her keenness. She tutored and encouraged her loyal recruits to take on tasks that they themselves had no idea they could accomplish. The wisest (maybe luckiest) decision of my life (and the moral of this tale) was to choose the most inspiring teacher to work with, rather than a merely interesting subject.

## Conflict of Interest Statement

The author declares that the research was conducted in the absence of any commercial or financial relationships that could be construed as a potential conflict of interest.
